# Importance of genetic cancer risk assessment as a strategy to stratify risk and provide precision prevention in high-risk patients and families

**DOI:** 10.1590/1806-9282.2024S117

**Published:** 2024-06-07

**Authors:** Patricia Ashton-Prolla, Maria Isabel Waddington Achatz, Miguel Angelo Martins Moreira, Edenir Inez Palmero, Diogo Cordeiro de Queiroz Soares, Victor Evangelista de Faria Ferraz, Inacelli Queiroz de Souza Caires, Rodrigo Santa Cruz Guindalini, Ana Carolina Leite Vieira Costa Gifoni

**Affiliations:** 1 Hospital de Clínicas de Porto Alegre, Medical Genetics Service – Porto Alegre (RS), Brazil.; 2 Universidade Federal do Rio Grande do Sul, Department of Genetics – Porto Alegre (RS), Brazil.; 3 Hospital Sirio-Libanês – São Paulo (SP), Brazil.; 4 Instituto Nacional de Câncer, Genetics Program – Rio de Janeiro (RJ), Brazil.; 5 Real Hospital Português, Oncogenetics Service – Recife (PE), Brazil.; 6 A.C. Camargo Cancer Center, Department of Oncogenetics – São Paulo (SP), Brazil.; 7 Universidade de São Paulo, Faculty of Medicine of Ribeirão Preto, Department of Genetics – Ribeirão Preto (SP), Brazil.; 8 Instituto D´Or de Pesquisa e Ensino – São Paulo (SP), Brazil.

## CANCER STATISTICS, HEREDITARY CANCER IN BRAZIL, AND THE CREATION OF THE BRAZILIAN NETWORK OF HEREDITARY CANCER (REBRACH)

Approximately 704,000 new cases of cancer are expected to occur per year in Brazil between 2023 and 2025, corresponding to an incidence of 222 per 100,000 in men and 186 per 100,000 in women. Data from the last decades show that an epidemiological transition is occurring and cancer will soon be the first cause of death by disease in Brazil^1,2^, overtaking cardiovascular diseases. Consequently, it is estimated that the annual cost of cancer treatment in the Brazilian public health system (SUS) will practically double until 2040, reaching R$ 7.8 billion^3^. Therefore, cancer prevention and early detection are key strategies to lower incidence, mortality growth, and costs of cancer care in the public health system. Although most cancers are associated with environmental causes, approximately 5–10% are mainly due to hereditary predisposition, characterized by multiple cases in a family, early age of onset, and frequently, diagnosis of multiple primary cancers in one person. Hereditary syndromes have been well-established for the most common solid tumors such as breast and colorectal cancers. However, recent expansion of genetic testing has identified hereditary versions of practically all types of cancers, associated or not with specific phenotypic criteria. In Brazil, several studies carried out in the last decades characterized the prevalence of germline pathogenic genetic variants associated with distinct cancer types including breast, colorectal, retinoblastoma, adrenocortical carcinoma, and ovarian cancers^4-8^. These studies showed a high prevalence of specific mutations also found in other populations as well as variants mostly or only observed in the Brazilian population^9^ and contributed to the growth of scientific knowledge in the field. They also helped insert oncogenetics and genetic cancer risk assessment (GCRA) in public and private health services and established in 2009 a network funded by the Brazilian National Council for Scientific and Technological Development (CNPq) and coordinated by the Brazilian National Cancer Institute (INCA) (Rede Nacional de Cancer Familial)^10,11^. This network is now an autonomous organization (www.rebrach.org.br) intended to promote actions to optimize access, health care assistance, training of health care professionals, and scientific investigations in hereditary cancer.

## IMPORTANCE OF IDENTIFYING INDIVIDUALS WITH INHERITED PREDISPOSITION TO CANCER

Inherited predisposition to cancer may be suspected by the presence of clinical features ("phenotype"), but nowadays, it may also be identified by genomic testing for other diseases (i.e., exome testing for pediatric disorders unrelated to cancer). Even when there is a phenotype suggesting hereditary cancer, germline genetic testing (molecular diagnosis) should be done to confirm the presence of a pathogenic variant in one or more of the hereditary cancer predisposition genes. Although these variants occur mostly in people with a suggestive phenotype, they may also be present in up to 26–56% of individuals who do not meet any clinical criteria. Several factors may explain this situation including: (1) individual from a small family, with few relatives, also known as limited family structure; (2) incomplete penetrance of most genes related to hereditary syndromes; (3) diagnosis of late-onset cancer; or (4) lack of knowledge about family history. The presence of a germline pathogenic or likely pathogenic variant in a cancer predisposition gene increases, often more than five times the lifetime risk of one or multiple cancers. Once a pathogenic or likely pathogenic variant is identified, regardless of the presence or absence of a characteristic phenotype, the carrier must be closely assessed for lifetime cancer risk and genetic counseling as well as cascade testing of other at-risk relatives is warranted.

## WHAT IS GENETIC CANCER RISK ASSESSMENT AND HOW IS IT DONE?

Genetic cancer risk assessment is the process of evaluating risk, identifying appropriate patients for genetic testing, reviewing the limitations, and determining the risks, benefits, and scope of testing. It is recommended before any genetic testing and should ideally also be performed after the test, especially if a positive result is found. It should be performed by a trained clinical geneticist or other health care professional who is experienced in cancer genetics. The most commonly used genetic testing approach nowadays is multi-gene panel testing (MGPT), which usually includes analysis of high and moderate penetrance genes and, in many cases, genes that are considered preliminary evidence, for which limited information is available about association with and/or causality for disease. MGPT increases the likelihood of identifying variants of unknown significance (VUS), and it also allows the identification of patients with more than one pathogenic variant. Despite the improved clinical utility of an expanded hereditary cancer gene panel, a higher rate of VUS is also expected which constitutes a challenge for counseling. Post-test counseling is an important part of the process, enabling discussions with the patient about the result itself, rationale for additional genetic testing in the patient or relatives (when appropriate), definition of cancer risks, and referrals (if necessary) for ongoing management. It is important to emphasize that counseling after genetic testing is key to discuss cancer risk for other family members and to provide clear recommendations about cascade testing of relatives.

In circumstances where genetic tests are not available, it is possible to use mathematical models to predict risks. Risk assessment tools often use models that combine personal health history information, family history, non-disease indicators of risk, and genetic/genomic data. However, the final confirmation of hereditary predisposition to cancer depends on the molecular diagnosis. The empiric risk models may help in the management of cases with a phenotype but no identifiable genetic variant after comprehensive genetic testing, a situation also known as "missing heritability."

## IMPORTANCE OF HIGH-QUALITY GENETIC TESTS IN THE IDENTIFICATION OF INDIVIDUALS WITH HEREDITARY CANCER

Germline genetic testing is offered by various laboratories, employing distinct approaches to assess sequence variants and large gene rearrangements. In recent years, laboratory practices have been rapidly evolving as a result of advancements in DNA and RNA analysis technologies, increased demand for genetic testing, and developments in the field of personalized medicine, where treatments are guided by test results. Therefore, when developing a molecular test for clinical diagnosis, it should meet certain minimum quality requirements, such as precision, accuracy, detection limits, and coverage. Furthermore, after designing and developing the test, validation studies must be conducted to ensure that the predefined performance has been achieved. In the report, identified variants need to be classified for their pathogenicity in accordance with internationally validated and updated guidelines, ensuring reproducibility in variant classification among different laboratories. Furthermore, the continuous deposition of detected variants in public databases by diagnostic laboratories will be essential for advancing the correct classification of potential pathogenicity and, consequently, the most appropriate clinical management for affected patients. Finally, other investigative avenues must often be pursued to assess potential pathogenicity or benignity, including functional assays in cellular and animal models and cosegregation analysis of variants in affected and unaffected family members, which is a process that may take several years until a final definition on pathogenicity is reached.

## WHAT CHANGES IN TERMS OF CANCER MANAGEMENT AND PREVENTION WITH GENETIC CANCER RISK ASSESSMENT?

The identification of hereditary cancer predisposition impacts various aspects of care, from risk reduction and screening recommendations to the planning of oncological treatment. It also enables genetic counseling tailored to reproductive issues, including preimplantation genetic diagnosis. This clinical utility, based on an increasingly robust literature, has positioned Oncogenetics as an integral part of the comprehensive medical care of oncologic patients. In addition to recommendations for a protective lifestyle (i.e., healthy diet, weight control, regular physical activity, and avoidance of alcohol and tobacco), high-risk management is based on differentiated recommendations for screening, chemoprophylaxis, and risk-reducing surgeries. Intensified screening is defined as screening beyond the level recommended for individuals at average risk. It includes adjustments to the recommended age of screening onset, the recommended screening intervals, and the methods involved in screening^12^. The screening protocol is defined taking into account individual-specific aspects, the diagnosed cancer predisposition syndrome, and the familial phenotype. For cancer risk reduction, evidence for chemoprophylaxis is still preliminary. Literature data suggest a protective role of tamoxifen or aromatase inhibitors for breast cancer^13^ and a potential role for aspirin in reducing the risk of colorectal cancer in patients with Lynch syndrome^14^. The role of other medications is still under development. In some scenarios, risk-reducing surgery may be the most appropriate approach. For example, in women with a hereditary predisposition to ovarian cancer associated with high-penetrance genes such as BRCA1 and BRCA2, the role of bilateral risk-reducing salpingo-oophorectomy (RRSO) is well established and reduces mortality^15^. In recent years, germline alterations have also gained value as predictive biomarkers for response to targeted systemic treatments. The best example of this is the use of poly(ADP-ribose) polymerase (PARP) inhibitors in patients with homologous recombination-deficient tumors, notably those with mutated BRCA1 and BRCA2. The success of this approach has so far led to the approval of four different PARP inhibitors for the treatment of several types of cancers, such as breast, ovarian, prostate, and pancreatic cancer^16^.

## COST-EFFECTIVENESS OF GENETIC TESTING AND PREVENTIVE STRATEGIES

Currently, the most studied cost-effectiveness models are conducted through analyses of hereditary breast/ovarian cancer panels, where the cost per quality-adjusted life-year (QALY) and the incremental cost-effectiveness ratio (ICER) are calculated, serving as a cost-effective screening measure in European countries and the United States, preventing cases of breast and ovarian cancer and avoiding deaths^17,18^. In Brazil, there are two studies of economic modeling of screen-and-treat strategies for women at risk of HBOC evaluating the implementation of BRCA1 and BRCA2 testing^19,20^. In both studies, Markov models with a lifelong time horizon were developed for a cohort of healthy women aged 30 years who fulfilled the criteria for testing according to the guidelines. Women who tested positive had several alternatives, including increased surveillance and the option of risk-reducing bilateral mastectomy and bilateral salpingo-oophorectomy. The BRCA1/BRCA2 genetic test and preventive strategies result in more QALYs and costs with an ICER of R$ 11,900–R$ 24,263/QALY. This ICER determined for BRCA1/BRCA2 genetic testing provision closely aligns with the cost-effectiveness threshold set forth by the World Health Organization (WHO) for low- and middle-income countries. Despite the absence of a stringent cost-effectiveness threshold in Brazil, the outcomes of this analysis advocate in favor of the implementation of BRCA1/BRCA2 testing among high-risk women in SUS.

## ACCESS TO GENETIC CANCER RISK ASSESSMENT IN THE BRAZILIAN HEALTH CARE SYSTEM (PUBLIC AND PRIVATE SECTORS)

The 2019 National Health Survey^21^ shows that around 71.5% of the Brazilian population has, as the only health care resource, the free, state-owned, and universal SUS. This proportion increases in the north and northeast of Brazil, reflecting historical inequities in the country's development. Additional private health insurance plan coverage was 26.0%, with the same great inequity between the Large Regions and Federation Units. The Southeast and South Regions emerged with the highest coverage proportional to their populations (34.9 and 30.5%, respectively). In Brazil, the clinical and laboratory assessment of hereditary cancer risk is under construction and reflects the challenges of health care for the Brazilian population. Within the scope of SUS, some public services, most linked to universities, philanthropic hospitals, or linked to foundations, established outpatient clinics for this purpose from the 1990s onward. Diagnostic resources, specifically molecular biology tests to investigate the presence of causative variants, are offered through research projects and other proprietary sources, as to date, they are not available in the public health system. In 2016, Ashton-Prolla and Seuanez^11^ highlighted that these resources covered less than 5% of the Brazilian population. This scenario has changed little since then. Supplementary Health has addressed this issue a little better, through its regulatory agency, the National Supplementary Health Agency (ANS). In 2013, the ANS published the first version of its list of procedures, accompanied by use guidelines for coverage of procedures in supplementary health care that includes, for the first time, cancer predisposition syndromes investigation. Currently, there are guidelines^22^ for Hereditary Breast and Ovarian Cancer Syndrome (HBOC), Lynch, and several other cancer predisposition syndromes. However, the guidelines adopted by ANS are restrictive and do not accommodate cancer-unaffected individuals, with a family history, except those who already have a relative with a detected mutation. Another restriction of these guidelines is the limitation on the use of multigene panel sequencing, favoring single-gene, sequential testing. Because of these restrictions, the ANS guidelines have become progressively more distant from the current approaches to testing proposed by several international medical societies.

## IMPORTANCE OF CAPACITY BUILDING AMONG HEALTH CARE PROFESSIONALS TO ENABLE GENETIC CANCER RISK ASSESSMENT AND ACCURATE IDENTIFICATION AND MANAGEMENT OF HIGH HIGH-RISK PATIENTS

Identification and management of hereditary cancer play an important role in identifying high-risk individuals and ensuring access to interventions that can prevent disease. However, to enable the benefits of the effective incorporation of genetic or genomic information into health care, a significant barrier to more equitable access to this technology is proper training of health care professionals at different levels^23^. In this sense, the limited availability of providers of GCRA is an important issue in most countries worldwide. Several professional societies have developed specific curricula and a few multidisciplinary short- and long-term training programs have been established^12,24^. Despite existing initiatives, there is an urgent need to invest in the education of health care providers to expand the number of providers available, reduce variability in knowledge regarding hereditary cancer, and qualify genetic service provision. The knowledge needed to provide accurate and comprehensive GCRA is complex and constantly changing. It requires training in several important domains: state of cancer genetics, state of genetic counseling and risk communication, state of technology of genetic testing and variant interpretation, and state of the art in terms of risk management including knowledge of the constantly changing treatment recommendations based on actionable inherited genetic variants. These professionals must also be equipped to handle the complex and rapidly evolving medical, technological, and ethical issues involved in the care of hereditary cancer patients and their relatives^25^. Therefore, capacity building should include training at different levels, from basic knowledge about the importance of identifying at-risk patients, need for referral and promoting this practice among peers (i.e., capacity-building interventions in primary care teams), to disease-specific training (i.e., in a specific tumor of a subset of tumors) to comprehensive training in all aspects of GCRA. Once training is complete, continuous education through periodic participation in multidisciplinary case discussions and tumor boards is also relevant to address the issue of rapidly evolving knowledge in the field. In Brazil, no formal qualification in GCRA, as proposed by international accreditation programs, exists to date for physicians or other health care professionals. For physicians, formal training in genetic counseling is offered only in medical genetics residency programs, but even among some of these, there is a lack of comprehensive and dedicated supervised training experience in cancer genetics. To approach this gap, two important initiatives are being developed by several professional societies led by the Brazilian Society of Medical Genetics and Genomics (SBGM). The first is the development of a 2000-h Theoretical and Practical Course in cancer genetics for physicians already approved by the Brazilian Medical Association (AMB) and currently under review by the Federal Council of Medicine (CFM). The second is the creation of a comprehensive genetic counseling training program for non-MD health care professionals sponsored by the Ministry of Health. Such initiatives are key to enhance adherence to and effectiveness of GCRA. They will also reduce the increasing harms related to lack of access to adequate genetic testing, inaccurate result interpretation, or failure to tailor cancer risk-reducing interventions appropriately.
